# Variceal Hemorrhage in Two Children With Congenital Heart Disease and Long-Term Pulmonary Venous Obstruction

**DOI:** 10.1097/PG9.0000000000000028

**Published:** 2020-12-03

**Authors:** Lay Queen Ng, Jonathan T.L. Choo, Marielle V. Fortier, Fang Kuan Chiou

**Affiliations:** From the * Gastroenterology, Hepatology and Nutrition Service, Pediatric Medicine, KK Women’s and Children’s Hospital, Singapore; † Cardiology Service, Department of Pediatric Subspecialties, KK Women’s and Children’s Hospital, Singapore; ‡ Department of Diagnostic and Interventional Imaging, KK Women’s and Children’s Hospital, Singapore.

## INTRODUCTION

Nonhepatic, cardiac-derived esophageal variceal hemorrhage is a rare condition which can arise from abnormal collateralization or shunting of blood from the bronchopulmonary circulation, typically in the setting of pulmonary venous obstruction (PVO) from congenital heart disease (CHD) (1). While evidence-based guidelines exist for the management of esophageal varices (EV) secondary to liver cirrhosis and portal hypertension (2), little is known about the natural history and appropriate management of cardiac-derived EV arising as a complication of CHD. We describe the clinical presentation and outcome of 2 cases of cardiac-derived EV hemorrhage in patients with CHD and long-standing PVO.

## CASE REPORT

### Case 1

Ten-year-old child with complex CHD (double inlet left ventricle, complete atrioventricular septal defect, malpositioned great arteries, hypoplastic right ventricle, post-surgical bidirectional cavopulmonary connection [BCPC] and resection of the left atrial cortriatriatum) presented with acute hematemesis and significant drop in hemoglobin from 21 to 18.9 g/dL. He had no evidence of liver disease/portal hypertension with normal transaminases and platelet counts (239 × 10^9^/L). There was no splenomegaly on physical examination and ultrasound imaging. He was not on anticoagulation therapy.

**FIGURE 1.**
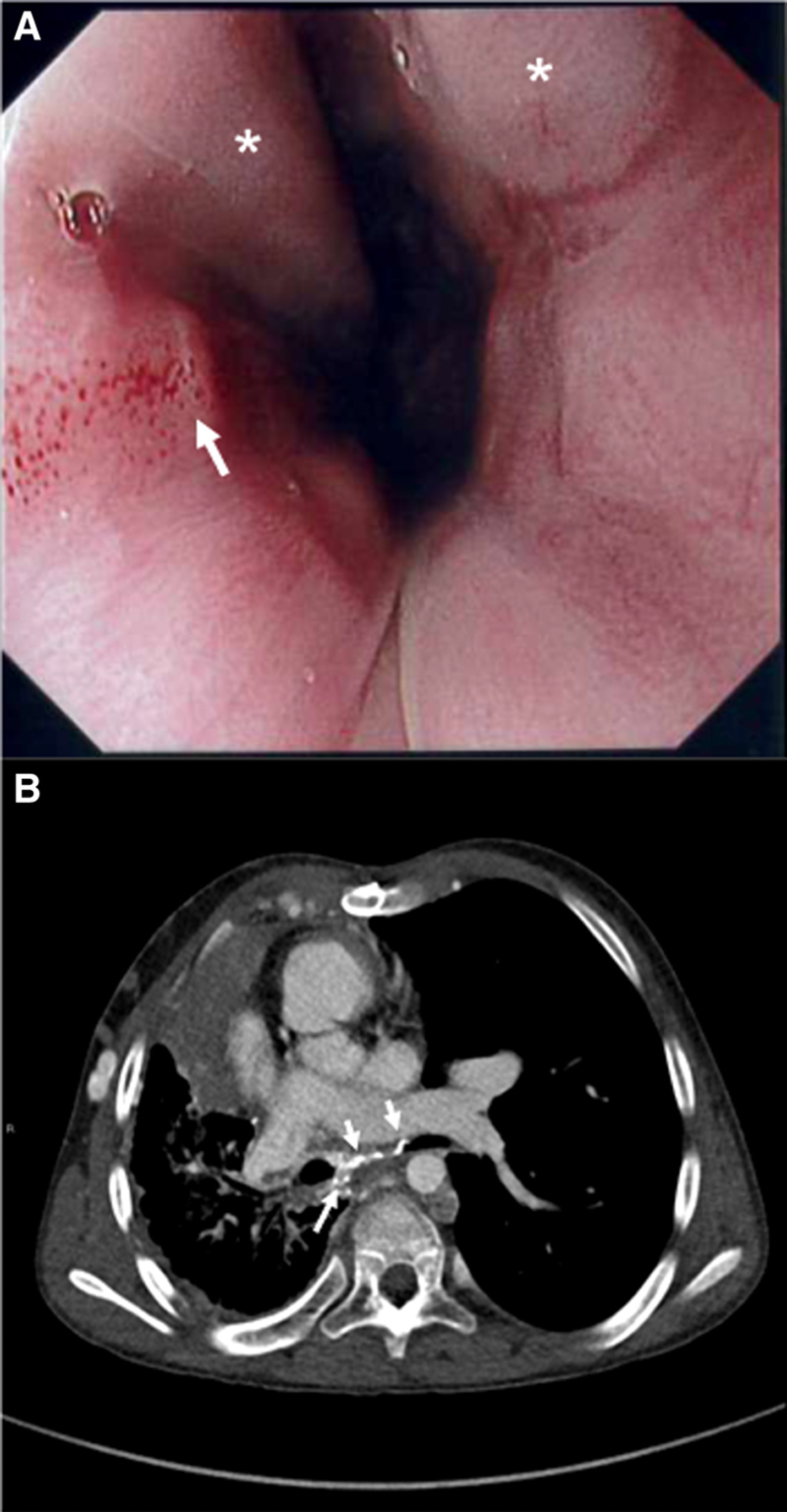
Composite image depicting cardiac-derived EV in patient 1. A, Endoscopic image demonstrating two columns of grade 3 esophageal varices (white asterisks) and red wale sign at the 9 o’clock position signifying active recent bleed (white arrow). B, Computer tomography angiogram of patient 1 demonstrating collaterals to left and right mainstem bronchus and surrounding the thoracic esophagus (arrows).

He underwent urgent upper gastrointestinal endoscopy, which showed grade 3 EV with stigmata of active bleeding (Fig. 1). Endoscopic variceal ligation (EVL) was performed with successful decompression of the varices; however, he was noted to develop moderate pulmonary hemorrhage through his endotracheal tube within minutes after EVL which spontaneously resolved. He presented again with hematemesis 5 days after EVL. This time he was managed successfully with conservative medical treatment comprising packed cell transfusions, empirical proton pump inhibitor (PPI), and octreotide with resolution of bleeding.

**FIGURE 2.**
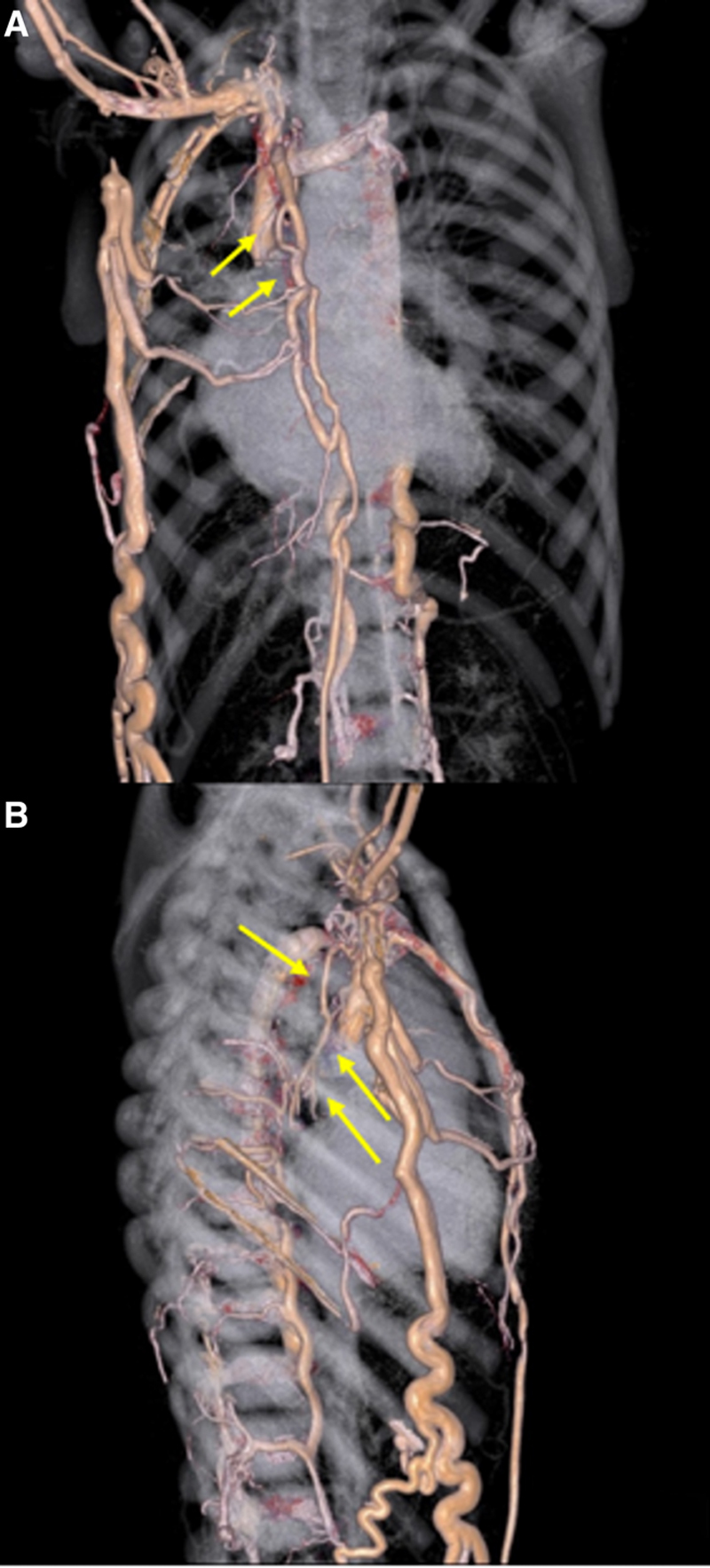
Extensive collateral network on CTA in patient 1. A, Three-dimensional computer tomography angiogram of the thorax from patient 1 showing extensive venovenous collaterals in the paraesophageal region (arrows). B, Lateral 3D image showing network of collaterals (arrows) surrounding mainstem bronchi and with tributaries to intercostals, anterior chest wall and azygous.

Computer tomography angiogram (CTA) showed PVO with extensive network of collateral vessels extending to the mainstem bronchus as well as to the paraesophageal veins that contributed to the formation of EV (Fig. 2). Further endoscopic reassessment and prophylactic EVL were deferred given concerns that obliterating these decompressing collaterals might redirect flow through the bronchopulmonary shunts and result in increased risk of pulmonary hemorrhage. He remained only on PPI therapy with no further EV hemorrhage in the 8 months of follow-up since.

### Case 2

A child with postoperative total anomalous pulmonary venous drainage, persistent PVO, and pulmonary hypertension, who had been on antiplatelet therapy, presented with the first episode of hematemesis at 18 months old. Hemoglobin (13.2 g/dL), platelet count (279 × 10^9^/L), liver enzymes, and coagulation screen were within normal limits. There was no hepatosplenomegaly and Doppler ultrasound confirmed normal hepatopedal flow within the portal vein. She was started on intravenous PPI and anticoagulation therapy was discontinued.

**FIGURE 3.**
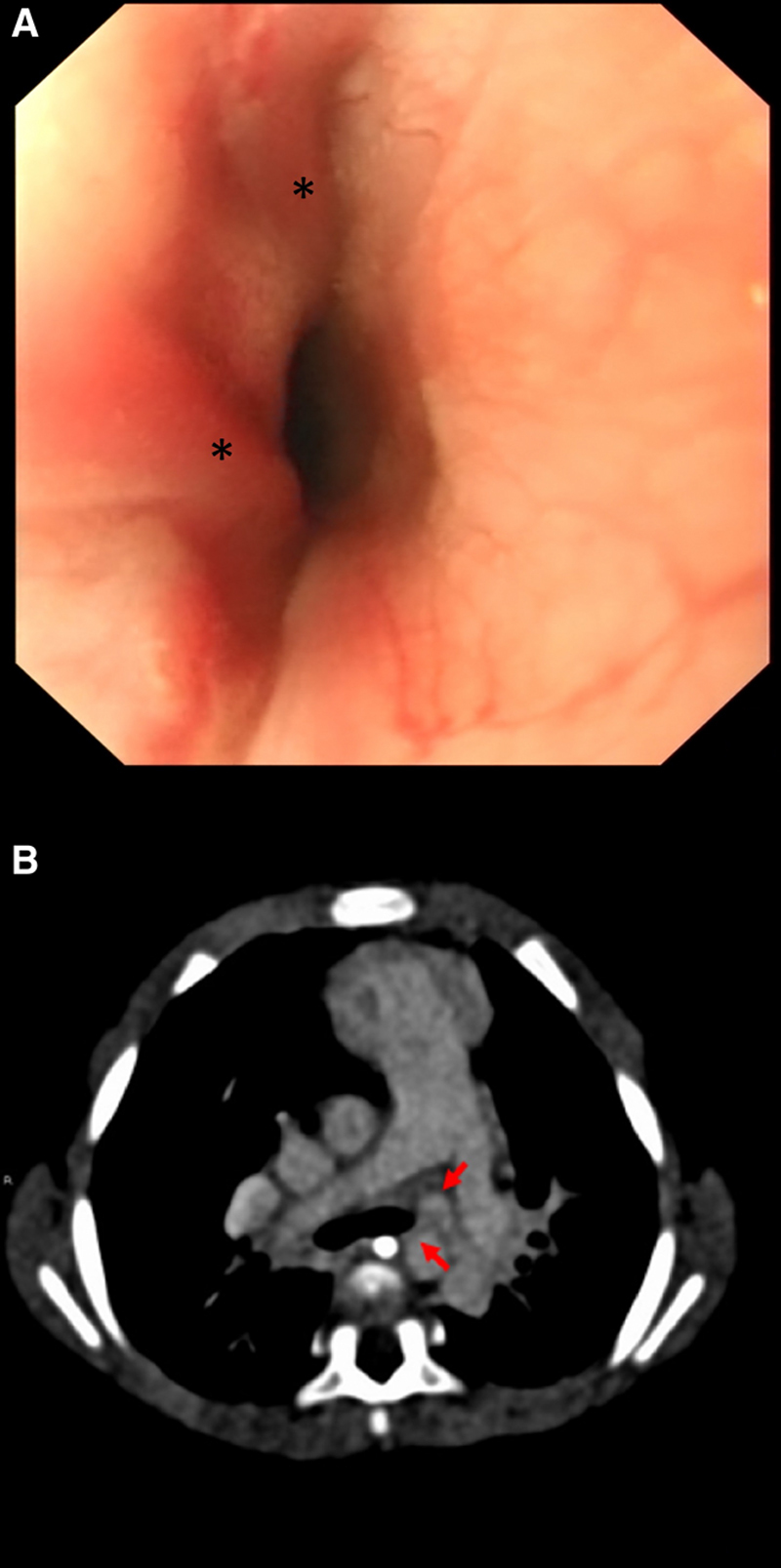
Composite image depicting cardiac-derived EV in patient 2. A, Endoscopic image showing grade 2 varices at 9 o’clock and 12 o’clock positions (black asterisks) in patient 2. B, Computer tomography angiogram from patient 2. Red arrows pointing to tangle of small collateral vessels from left pulmonary hilum to paraesophageal region, seen on delayed views.

Urgent upper endoscopy demonstrated the presence of grade 2 EV but no active bleeding. As there was no other lesion found in the stomach and duodenum, the bleeding source was assumed to be from EV which had spontaneously stopped bleeding. No endoscopic intervention was performed given the absence of active bleeding. CTA and cardiac catheter study confirmed the underlying PVO and demonstrated evidence of collateralization between pulmonary veins and esophageal veins (Fig. 3). Hepatic venous pressure gradient was measured to be low-normal at 1 mmHg consistent with the absence of underlying portal hypertension/liver disease. The cardiac catheter study was technically difficult and attempts to dilate and stent the left upper pulmonary vein were unsuccessful.

Despite having had no further intervention, she had no recurrence of EV hemorrhage in the 2 years follow-up while continuing on PPI and diuretics.

## DISCUSSION

The formation of collateral vessels is well-described in patients with congenital cardiac malformations both before and after surgery. Collateral vessels arise in response to hypoxemia or a pressure difference between two areas in the circulatory system and may result from neovasculogenesis (3) or from the opening of embryonic venous channels. In particular, collateral vessels are seen in patients with a single ventricle physiology after BCPC surgery in 2 forms: Aortopulmonary collaterals or venovenous collaterals, with an estimated prevalence of between 20.2% and 36% (4,5).

The entity of cardiac-derived EV resulting from the presence of these collaterals is rare, with an unknown incidence and limited case reports (6,7). Although underlying cardiac anatomy differed in our 2 patients, both had pulmonary hypertension resulting from PVO, supporting the hypothesis that the formation of unusual anastomotic connections between the pulmonary and systemic venous circulations resulted in EV formation. The development of these collateral channels allowed blood flow from high-resistance pulmonary venous systems to the low-pressure esophageal circulation, effectively decompressing the pulmonary circulation but unfortunately also predisposing to the formation of EV with the risk of variceal hemorrhage.

The first step in the diagnostic approach is to exclude an underlying primary liver pathology. Both cases had no underlying liver pathology or clinical signs of portal hypertension as evident by means of normal liver function tests, platelet counts, and the absence of splenomegaly. Echocardiography should be performed to evaluate cardiac function, assess for atrioventricular valve regurgitation, and exclude PVO. CTA is an important diagnostic modality to demonstrate the collateral vessels giving rise to cardiac-derived EV. In our experience, a customized protocol using the test bolus technique and accurate timing of delayed scans improved the diagnostic yield in demonstrating these abnormal collaterals vessels in question.

Current established indications and risk–benefit data on EVL in liver cirrhosis and portal hypertension cannot be applied in patients with cardiac-derived EV. In patients with PVO, the formation of collaterals and shunts has an effect in partial reduction of pulmonary pressure, and obliterating these cardiac-derived EV may potentially result in the worsening of pulmonary hypertension and pulmonary hemorrhage as seen in our experience with Case 1. No complication was encountered in Case 2, although we acknowledge that it is difficult to predict the outcome had she similarly undergone EVL.

Therefore, for cardiac-derived EV, we propose that EVL be indicated only in the acute setting of significant EV hemorrhage that is refractory to medical therapy. In the absence of active bleed, surveillance endoscopy and prophylactic EVL should not be routinely recommended. For cases with refractory or recurrent EV bleeds, definitive surgical interventions such as pneumonectomy, or endovascular therapy such as stenting or embolization may be considered. Miller et al (7) described the eventual resolution of EV after right pneumonectomy, while Harrison et al (8) described a successful resolution of hemoptysis and EV bleed after percutaneous vascular coil occlusion of collaterals in an adult patient with unilateral pulmonary vein atresia.

In conclusion, we highlight the importance of recognizing nonhepatic, cardiac-derived EV, which is a rare complication in patients with complex CHD; EVL for cardiac-derived EV may be associated with risk of significant pulmonary complications.
